# Neurological Involvement in Multisystem Inflammatory Syndrome in Children: Clinical, Electroencephalographic and Magnetic Resonance Imaging Peculiarities and Therapeutic Implications. An Italian Single-Center Experience

**DOI:** 10.3389/fped.2022.932208

**Published:** 2022-08-12

**Authors:** Stefania Maria Bova, Ludovica Serafini, Pietro Capetti, Andrea Riccardo Dallapiccola, Chiara Doneda, Arianna Gadda, Luisa Lonoce, Alessandra Vittorini, Savina Mannarino, Pierangelo Veggiotti, Milan MIS-C Study Group, Sara Olivotto

**Affiliations:** ^1^Pediatric Neurology Unit, V. Buzzi Children’s Hospital, ASST Fatebenefratelli Sacco, Milan, Italy.; ^2^Department of Biomedical and Clinical Science, University of Milan, Milan, Italy.; ^3^Department of Pediatrics, V. Buzzi Children’s Hospital, ASST Fatebenefratelli Sacco, Milan, Italy.; ^4^Pediatric Intensive Care Unit, V. Buzzi Children’s Hospital, ASST Fatebenefratelli Sacco, Milan, Italy.; ^1^Pediatric Neurology Unit, V. Buzzi Children’s Hospital, ASST Fatebenefratelli Sacco, Milan, Italy; ^2^Department of Biomedical and Clinical Science, University of Milan, Milan, Italy; ^3^Department of Paediatric Radiology and Neuroradiology, V. Buzzi Children’s Hospital, Milan, Italy; ^4^Faculty of Medicine and Surgery, University of Milan, Milan, Italy; ^5^Department of Pediatrics, V. Buzzi Children’s Hospital, Milan, Italy; ^6^Pediatric Cardiology Unit, V. Buzzi Children’s Hospital, Milan, Italy

**Keywords:** COVID-19, SARS-CoV-2, multisystem inflammatory syndrome in children (MIS-C), acute immune-mediated encephalitis in children, neurological involvement in MIS-C, cytotoxic lesion of the corpus callosum (CLOCC), therapy of MIS-C

## Abstract

**Objective:**

To describe neurological involvement in multisystem inflammatory syndrome in children (MIS-C) and to evaluate whether neurological manifestations are related to the degree of multiorgan involvement and inflammation.

**Methods:**

The authors conducted a retrospective analysis of clinical, electroencephalographic (EEG), neuroradiological (MRI), and CSF parameters in 62 children with MIS-C (45 M, age 8 months—17 years, mean age 9 years) hospitalized between October 1, 2020 and March 31, 2022.

**Results:**

Neurological involvement was documented in 58/62 (93.5%) patients. Altered mental status was observed in 29 (46.7%), focal neurological signs in 22 (35.4%), and non-specific symptoms in 54 (87%). EEG was performed in 26/62 children: 20 showed EEG slowing, diffuse or predominantly over the posterior regions. Ten patients underwent brain MRI: three showed a cytotoxic lesion of the corpus callosum. CSF analysis, performed in six patients, was normal. On the basis of the clinical and EEG findings, two profiles of neurological involvement were identified: 16/62 (26%) patients presented encephalitis with rapid-onset encephalopathy, focal neurological signs, and EEG slowing; 42/62 (68%) showed mild neurological involvement with mild or non-specific neurological signs. All patients received intravenous immunoglobulin and methylprednisolone (MTP), low-molecular-weight heparin, and therapeutic-dose anticoagulant treatment. Children with severe encephalopathy received intravenous MTP at 30 mg/kg/day for 3 days, obtaining rapid clinical and EEG improvement. Neurological assessment at discharge was normal in all cases. Children with encephalitis were younger than those without (median age 5 and 10 years, respectively); no differences between the two groups were found in the other parameters: comorbidities, fever, number of organs and systems involved, shock, hospitalization, pediatric intensive care unit admission, non-invasive ventilation, inotropic support, laboratory data.

**Conclusion:**

Neurological involvement in MIS-C is frequent but not serious in most cases: around two thirds of the affected children had mild and short-lasting symptoms. It seems to be related to age, but not to the degree of multiorgan involvement and inflammation. In children with acute immune-mediated encephalitis, the clinical picture was dominated by encephalopathy that disappeared with immunomodulatory therapy. Neurological assessment allowed timely diagnosis and treatment.

## Introduction

Although the primary target of SARS-CoV-2 is the respiratory system, symptoms of nervous system involvement are so frequent in infected patients that some of them—ageusia and anosmia—are considered pathognomonic of COVID-19. The central nervous system (CNS) damage occurring in this setting may be due to direct infection of brain vascular endothelial cells or of the olfactory nerve, or indirect infection resulting from para or postinfectious inflammation, triggered by cytokine storm effects on blood-brain barrier (BBB) permeability (1, 2). Different neurobiological processes and mechanisms may underlie the link between SARS-Cov-2 and COVID-19 in the brain. These pathophysiological mechanisms lead to specific clinical pictures and neurological signs and symptoms that appear in sequence, although they sometimes overlap considerably. The first neurological sign is loss of smell or taste due to the SARS-Cov-2 infection of the epithelial cells of the nasal and oral mucosa, and to retrograde transport from the nasal mucosa to the brain, in the presence of a low and controlled cytokine storm. The next neurological symptoms, corresponding to stage two of neuroCOVID (from the second week after the onset of symptoms), reflect the activation of a robust immune response characterized by high cytokine, ferritin, C-reactive protein, and D-dimer levels, a hypercoagulable state that could result in strokes, and a heightened immune response, which also causes cerebral vasculitis. Finally, the third stage (from the fourth week after the onset of symptoms) includes damage to the BBB and infiltration of cytokines, blood components and viral particles into the brain parenchyma, possibly leading to delirium, encephalopathy and seizures (2).

In adults, neurological involvement is reported in around 30–40% of patients, and encephalopathy, induced by hypoxia or systemic diseases, is common. Cerebrovascular diseases are recognized as primary neurological complications and have been associated with a worse outcome (1). The spectrum of neurological involvement in children and adolescents, on the other hand, is still unclear, both in acute COVID-19 and in multisystem inflammatory syndrome (MIS-C), the severe hyperinflammatory disease that has been documented in some pediatric patients who have been exposed to SARS-CoV-2 (3–5). Shock, which many older MIS-C patients experience, can also be a factor in CNS involvement (6).

Although children are largely spared the severe acute effects of SARS-CoV-2 infection, MIS-C, albeit rare, is a severe disease that affects multiple organs including the CNS. Its pathophysiological mechanism is still unclear, but it is thought to be a highly complex postinfectious phenomenon resulting in hyperinflammation (7). MIS-C is underlain by a series of events very similar to those underlying the most serious neurological manifestations of SARS-CoV-2 infection described above, i.e., neuroCOVID stages two and three (2). Acute SARS-CoV-2 infection triggers a proinflammatory reaction and, in a genetically susceptible child, a delayed hyperinflammatory reaction consisting of vasculitis with augmented levels of lymphocyte T-helper 17 and T-helper 1 mediators, and a cytokine storm, including massive release of inflammatory mediators and exaggerated activation of the immune system leading to BBB damage (1). MIS-C was first described in April 2020 in Europe, but it is now reported and documented worldwide (8). Its true incidence is unknown. A recent US paper reported an adjusted estimated incidence of 1–10 cases per 1,000,000 people per month (9).

To date, the largest published study dealing with neurological involvement in children with SARS-CoV-2 infection is a retrospective study of a population of 1,695 patients; all were younger than 21 years and were hospitalized for MIS-C (in 616 cases) or acute COVID-19, between March and December 2020. Of these patients, 365 (22%) developed neurological complications (3). The vast majority showed transient symptoms, while 12% had a severe clinical presentation, which could consist of focal CNS disease (acute ischemic or hemorrhagic stroke, cerebral venous sinus thrombosis, or focal cerebral arteriopathy) or widespread CNS involvement with severe encephalopathy (CNS infection, acute disseminated encephalomyelitis, encephalopathy with white matter and corpus callosum lesions, or acute fulminant cerebral edema), or peripheral nervous system disorders (Guillain-Barré syndrome and variants). Although the mechanisms underlying the CNS damage were diverse, inflammation was found to be more serious in patients who developed severe neurological complications. Approximately one in four patients with neurological involvement presented altered awareness or confusion. The distribution of other symptoms was age related: seizures and status epilepticus were most commonly seen in younger patients, whereas anosmia and/or ageusia, headache, and fatigue/weakness were most commonly found in older patients. In these patients, severe sequelae were not rare: 26% of patients with neurological involvement died and 40% survived with a new neurological deficit.

Sa et al. in a retrospective review, reported neurological involvement in 9/75 children with MIS-C (12%). Two children developed cerebrovascular disease, and seven presented encephalopathy, in one case associated with hippocampal and splenium of corpus callosum changes. One child with extensive stroke died, and of the surviving eight children, half presented neurological sequelae at the 3-month follow up. Children with neurological symptoms were found to have significantly higher systemic inflammatory markers than children without. MIS-C-associated neurological involvement seems to be linked to a systemic para or postinfectious immune-mediated phenomenon with a distinct immunophenotype characterized by high levels of interleukin activation (10, 11).

Our research team also previously described acute encephalitis in a series of seven children with MIS-C. These patients displayed rapid-onset encephalopathy with drowsiness, irritability, mood deflection, focal neurological signs, and specific EEG abnormalities. MRI and CSF were normal. In all cases, the clinical picture rapidly improved with intravenous immunoglobulin therapy (IV IG) and high-dose intravenous (IV) methylprednisolone (MTP), and EEG normalized within 2 weeks of the neurological recovery. The severity and duration of the EEG abnormalities was proportional to the extent of the neurological involvement (5).

Abel et al., in 2020, reported the case of a previously healthy 2-year-old child with reversible encephalopathy with EEG slowing and bilateral thalamic lesions. He was successfully treated with IV steroids and IVIG, and discharged after 15 days on oral steroids, with mild residual weakness requiring physical therapy (4).

Hilado et al. recently reported the cases of three children with SARS-CoV-2 antibodies (but no multisystem involvement) who presented postinfectious autoimmune-mediated encephalitis and showed a good outcome after high-dose IV MTP treatment (12).

Overall, the currently available data show that children with MIS-C may present various neurological complications, some with a severe prognosis, and suggest that the spectrum of neurological involvement in this syndrome could be wider and more complex than is currently thought. Furthermore, since the clinical picture of MIS-C tends to be dominated, in particular, by systemic and cardiac complications, it is reasonable to think that signs of neurological involvement may be missed in many cases. Indeed, it seems likely that patients with mild and non-specific neurological symptoms are not adequately investigated, while neurological signs and symptoms appearing in those with multiorgan failure may be ignored, misunderstood, or underestimated.

Conversely, in our hospital neurological assessment has recently become an integral part of the multidisciplinary workup and management protocol applied in children admitted with a definite diagnosis of MIS-C. In the present study, we set out to describe neurological involvement in patients with MIS-C, characterizing its profile and severity in order to define the characteristics of subjects with more severe pictures, warranting a more aggressive immunomodulatory therapy, and to evaluate whether neurological involvement is related to the degree of multiorgan involvement and the inflammatory state.

## Materials and Methods

### Patients

The study includes all children and adolescents (aged ≤ 18 years) consecutively hospitalized at the Pediatric Department of Vittore Buzzi Children’s Hospital, a tertiary referral pediatric hospital in Milan, between October 1, 2020 and March 31, 2022, with a diagnosis of MIS-C meeting the relevant WHO criteria and American College of Rheumatology recommendations (10). Children with mimicking conditions (Kawasaki Disease, Toxic Shock Syndrome, Bacterial Sepsy, Macrophage Activation Syndrome, Myocarditis) were excluded.

For all patients the following data were recorded:

- general demographic and clinical data, comorbidities.
- clinical presentation: duration of fever, presence of organ and system involvement (neurological, cardiological, abdominal, respiratory, renal, mucocutaneous), shock.
- duration of hospitalization.
- pediatric intensive care unit (PICU) admission, non-invasive ventilation support (NIV), inotropic support.
- laboratory data: levels of white blood cells, neutrophils, lymphocytes, platelets, hemoglobin, C-reactive protein, ferritin, D-dimer, N-terminal proB-type natriuretic peptide (NTproBNP), and troponin T (high sensitivity).

The study was conducted according to the guidelines of the Declaration of Helsinki and approved by the Institutional Review Board of the Vittore Buzzi Hospital (Protocol n. 2021/ST/004). All participants or their legal guardians were asked for and gave their written consent after being informed about the nature of the study.

### Neurological Clinical and Instrumental Workup

**FIGURE 1:**
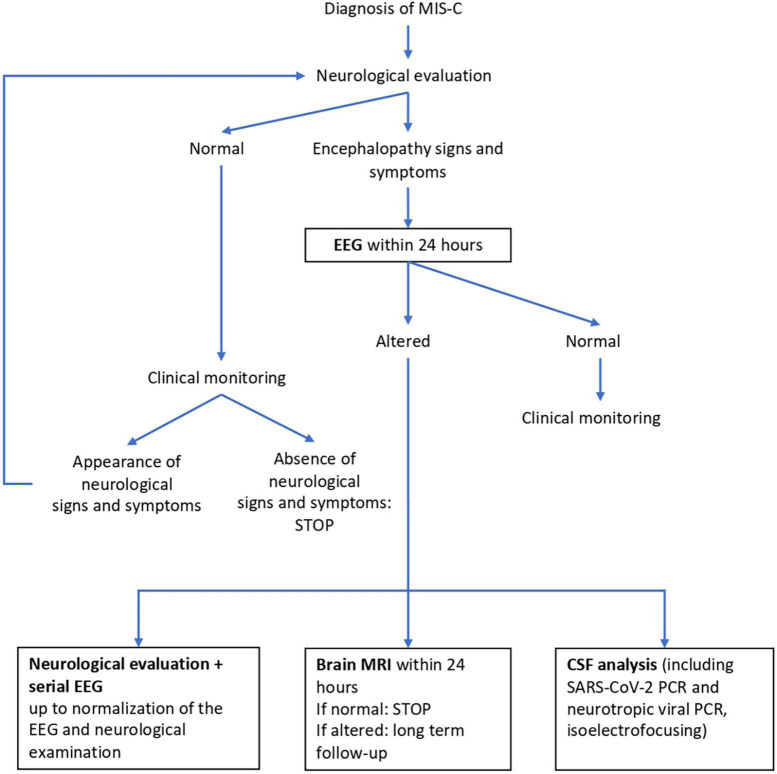
Neurological management algorithm. EEG, electroencephalography; MRI, magnetic resonance imaging; CSF, cerebro spinal fluid.

In our hospital, neurological clinical assessment is part of the diagnostic workup of children diagnosed with MIS-C. It is currently carried out both at admission and during the hospital stay, in accordance with our previously reported assessment protocol, also described below (4). Children who experienced shock had stable hemodynamic conditions when undergoing neurological assessment. Figure 1 shows our patient management algorithm. Clinical and instrumental data are collected in a dedicated database.

#### Neurological Clinical Assessment

The clinical assessment is carried out by a child neurologist in all children with a confirmed diagnosis at hospital admission. Signs and clinical symptoms are classified as:

*- signs of altered mental status* (altered state of consciousness, irritability or agitation, behavioral changes, i.e., emotional lability/impulsivity, mood deflection/anxiety);

*- focal neurological signs* (abnormal eye movements, facial asymmetries, gait disturbances, hemiparesis/hemiplegia, flaccid paralysis, dyskinesia, myoclonias, changes in speech, memory deficits, visual/auditory hallucinations, seizures, rigor nucalis, photophobia, muscle tone alterations, abnormal reflexes);

*- non-specific symptoms* (apathy, lack of appetite, asthenia, changes in the sleep/wake rhythm, headache, limb or trunk pain, paresthesia/anesthesia).

***EEG*** is performed, in both wakefulness and sleep, in subjects presenting signs and symptoms suggestive of CNS involvement, i.e., altered mental status and neurological signs. EEG abnormalities are classified as focal or diffuse according to the characteristics of the background activity slowing or the presence and localization of epileptiform changes and periodic and rhythmic patterns.

***CSF analysis*** (including SARS-CoV-2 PCR and neurotropic viral PCR, isoelectrofocusing) is performed in subjects with severe encephalopathy.

***Brain MRI*** is performed in subjects with severe encephalopathy.

***Profiles of neurological involvement*** were identified on the basis of clinical and EEG findings. Patients were divided into two groups accordingly:

***Encephalitis***, defined according to the International Encephalitis Consortium (13), comprising two subgroups:

- severe encephalopathy: patients who presented altered mental status + three or more focal neurological signs + diffuse EEG abnormalities;
- moderate encephalopathy: patients with altered mental status + one or two focal neurological signs + focal EEG abnormalities;

***Mild neurological involvement***, comprising two subgroups:

- mild clinical involvement: patients with mild signs of altered mental status or one or two focal neurological signs.
- non-specific neurological signs: patients with only non-specific neurological findings.

### Therapy

All the patients were treated according to our internal protocol (14). The rationale of the therapy is to counter the hyperimmune response that characterizes the disease. Accordingly, the cardinal treatment consisted of IVIG 2 g/kg and IV MTP 2 mg/kg for 5 days. In the presence of significant oxygen requirement, mild organ injury, and/or moderately reduced left ventricular ejection fraction (LVEF), MTP 10 mg/kg for 1 day then 2 mg/kg for 5 days was given before tapering over a period of 2 weeks. In patients needing respiratory or inotropic support, in the presence of moderate to severe organ damage, and in children with encephalitis with severe encephalopathy, we used a high MTP dose (30 mg/kg) for 3 days followed by 2 mg/kg for 5 days before tapering over a period of 2 weeks.

Anti-thrombotic prophylaxis was started in all patients > 12 years old, and was considered in those < 12 years old if the D-dimer level was high (>5 times the upper normal value) or if there was at least one known risk factor for thromboembolism. Anticoagulation therapy was prescribed in the presence of thrombosis or severe left ventricular (LV) dysfunction. Once the D-dimer level fell or LV function normalized, heparin was replaced with low-dose aspirin for 3–4 weeks.

### Statistical Analysis

Quantitative values are described as the mean and standard deviation (SD), or the median and interquartile range if not normally distributed (Shapiro–Wilk test). Qualitative variables are described as counts and percentages. Comparisons between groups were made with a chi-square test or Fisher’s exact test for qualitative variables, and with a *t*-test or Mann–Whitney test for quantitative data.

## Results

### General and Non-neurological Data

Demographics and clinical characteristics, comorbidities, clinical presentation, and laboratory data are reported in Table 1. The sample consisted of 62 patients (45 M, 72.5%; age 8 months—17 years, mean age 9 years). No preexisting major common systemic comorbidities were recorded. Sixteen patients (25.8%) were overweight; none were obese. None of the patients had congenital heart disease or preexisting cardiovascular disease.

**TABLE 1:** Characteristics of 62 patients (age < 18 years) hospitalized for MIS-C.

		All	Encefalopathy	Mild/No neurological involvement	*P*-value (<0.05)
**Patient number**, n (%)		62 (100)	16 (25.8)	46 (74.2)	
**Age** (y), median (range)		9 (0.8–17)	5 (2–10)	10 (0.8–17)	0.00048
**Gender**, n (%)					
	**Male, n (%)**	45 (72.5)	11 (68.75)	34 (73.9)	0.74
	**Female, n (%)**	17 (27.5)	5 (31.25)	12 (26.1)	0.74
**Ethnicity**, n (%)					
	**Caucasian, n (%)**	45 (72.5)	13 (81.25)	32 (69.6)	0.51
	**African, n (%)**	6 (9.7)	2 (12.5)	4 (8.7)	0.64
	**Asian, n (%)**	5 (8.1)	1 (6.25)	4 (8.7)	1
	**Hispanic, n (%)**	6 (9.7)	0 (0)	6 (13)	0.32
**Risk factors for neurological involvement**					
	Pre-existing neurological disorders (*), n (%)	9 (14.5)	3 (18.75)	6 (13)	0.68
	Pre-existing immunological disorders (**), n (%)	8 (12.9)	4 (25)	4 (8.7)	0.18
	**Overweight, n (%)**	16 (25.8)	3 (18.75)	13 (28.25)	0.73
**Inflammation**					
	**Serology positive, n (%)**	62 (100)	16 (100)	46 (100)	1
	**Positive RT-PCR, n (%)**	62 (100)	16 (100)	46 (100)	1
	**Fever, n (%)**	62 (100)	16 (100)	46 (100)	1
	**Fever duration (days), median (range)**	7 (2–14)	7 (4–14)	7 (2–14)	0.61
**Organs/systems involved**					
	**Neurological involvement, n (%)**	58 (93.5)	16 (100)	42 (91.3)	0.56
	**Cardiological involvement, n (%)**	19 (30.65)	5 (31.25)	14 (30.4)	1
	**Abdominal involvement, n (%)**	52 (83.9)	13 (81.25)	39 (84.8)	0.7
	**Respiratory involvement, n (%)**	41 (66.1)	13 (81.25)	28 (60.9)	0.22
	**Acute kidney injury, n (%)**	0 (0)	0 (0)	0 (0)	1
	**Mucocutaneous involvement, n (%)**	46 (74.2)	11 (68.75)	35 (76.1)	0.74
	**2 organs/systems involved, n (%)**	29 (46.8)	6 (37.5)	23 (50)	0.56
	** > 2 organs/systems involved, n (%)**	33 (53.2)	10 (62.5)	23 (50)	0.56
	**Shock, n (%)**	10 (16.1)	2 (12.5)	8 (17.4)	1
**Hospitalization**					
	**Duration of hospitalization (days), median (range)**	12 (7–26)	13 (9–20)	12 (7–26)	0.66
	**PICU admission, n (%)**	36 (58)	7 (43.75)	29 (63)	0.24
	**PICU stay (days), median (range)**	2 (0–12)	1 (0–12)	2.5 (0–9)	0.92
	**NIV, n (%)**	15 (24.2)	2 (12.5)	13 (28.25)	0.31
	**NIV stay (days), median (range)**	0 (0–6)	0 (0–6)	0 (0–6)	0.67
	**Inotropic support, n (%)**	16 (25.8)	5 (31.25)	11 (23.9)	0.74
	**Therapeutic-dose anticoagulation, n (%)**	12 (19.3)	4 (25)	8 (17.3)	0.65
**Laboratory data**					
	**WBC (10^3^/mmc), median (range)**	8,685 (2,226–33,500)	8,990 (3,420–33,500)	8,400 (2,226–30,850)	0.99
	**N (10^3^/mmc), median (range)**	6,660 (1,520–27,805)	7,255 (1,810–27,805)	6,580 (1,520–27,010)	0.25
	**L (10^3^/mmc), median (range)**	800 (230–8,610)	995 (230–4,310)	780 (280–8,610)	0.3
	**PLT (10^3^/mmc), median (range)**	168 (27–398)	181 (75–339)	162.5 (27–398)	0.78
	**Hb (g/dl), median (range)**	11.2 (7.5–14.6)	10.95 (8.2–14.1)	11.4 (7.5–14.6)	0.71
	**PCR (mg/l), median (range)**	168.05 (18.6–456)	170 (24.3–265)	165.65 (18.6–456)	0.15
	**Ferritin (mcg/l), median (range)**	505.5 (96–5,389)	430 (221–2,245)	606 (96–5,389)	0.18
	**D-Dimer (mcg/l), median (range)**	2,795 (347–25,385)	3,808 (1,114–23,000)	2,777 (347–25,385)	0.46
	**NT-Pro BNP (ng/l), median (range)**	3757.5 (73–35,000)	3,406 (73–35,000)	3757.5 (167–35,000)	0.4
	**Tnt (ng/l), median (range)**	34.5 (2–1,342)	14 (3–820)	44.5 (2–1,342)	0.23

*Epilepsy, neuromuscular disease, cerebral palsy, extreme prematurity (24 weeks), febrile seizures, psychomotor delay, specific learning disabilities, language disorders.

**Autoimmune thyroiditis, autoimmune arthritis, autoimmune dermatitis, allergy. PICU, pediatric intensive care unit admission; NIV, non-invasive ventilation; WBC, white blood cells; N, neutrophils; L, lymphocytes; PLTs, platelets; Hb, hemoglobin; CRP, C-reactive protein; NT-ProBNP, amino-terminal fragment of the brain natriuretic peptide; TnT, troponin T.

Nine (14.5%) patients had neurological comorbidities (epilepsy, neuromuscular disease, febrile convulsions, cerebral palsy, neurodevelopmental disorder, severe prematurity), while 8 patients (12.9%) had a previous history of immunological diseases (allergies, autoimmune thyroiditis, arthritis, dermatitis). Fever was present in all the patients, with a median duration of 7 days at diagnosis. Laboratory testing (Table 1) showed elevated inflammatory markers (C-reactive protein, fibrinogen, ferritin, D-dimer levels), neutrophilia with lymphopenia, and increased cardiac biomarkers. In 33 children (53.2%), more than two organs were involved, excluding the CNS. All 62 patients had positive IgG serology for the virus, and were treated with IVIG at the time of diagnosis, with a median delay of 5 ± 2 days from the onset of symptoms. Steroid (IV MTP) treatment was added in 58 patients (93.5%): 9 (14.5%) received the highest dose, 13 (21%) the intermediate dose, and 36 (58%) the lowest dose, as defined by our protocol. Low-molecular-weight heparin was prescribed in 62 patients (100%), of whom 12 (19.3%) received therapeutic-dose anticoagulant treatment. All the patients were discharged under treatment with low-dose aspirin. Thirty-six patients (58%) were admitted to the PICU. No patients required intubation and mechanical ventilation, while 15 (24.2%) needed NIV. Hospitalization for MIS-C lasted an average of 12 days (7–26 days).

### Neurological Clinical and Instrumental Workup

#### Neurological Clinical Assessment

Neurological involvement was documented in 58/62 (93.5%) patients. Table 2 shows the neurological findings, both in the entire sample and by severity of neurological involvement.

**TABLE 2:** Neurological presentation/involvement and therapy of 62 patients (age < 18 years) hospitalized for MIS-C.

			Total n 62 (100)	Severe n 7 (11.3)	Moderate n 9 (14.5)	Milld n 17 (27.4)	Non-specific n 25 (40.3)	No neurological symptoms n 4 (6.5)
Neurological symptoms	Signs of altered mental status	1. Altered state of consciousness	16 (25.8)	5 (71.4)	6 (66.6)	5 (29.4)	0 (0)	0 (0)
		2. Irritability/agitation	18 (29)	6 (85.7)	8 (88.8)	4 (23.5)	0 (0)	0 (0)
		3. Behavioral changes	10 (161)	5 (71.4)	2 (22.2)	3 (17.6)	0 (0)	0 (0)
		4. Mood deflection/anxiety	9 (14.5)	2 (28.5)	1 (11.1)	6 (35.2)	0 (0)	0 (0)
	Focal signs	5. Abnormalities of eye movements	3 (4.8)	1 (14.2)	0 (0)	2 (11.7)	0 (0)	0 (0)
		6. Facial asymmetries	1 (1.6)	0 (0)	0 (0)	1 (5.8)	0 (0)	0 (0)
		7. Gait disturbance	8 (12.9)	4 (57.1)	4 (44.4)	0 (0)	0 (0)	0 (0)
		8. Hemiparesis/hemiplegia	0 (0)	0 (0)	0 (0)	0 (0)	0 (0)	0 (0)
		9. Flaccid paralysis	0 (0)	0 (0)	0 (0)	0 (0)	0 (0)	0 (0)
		10. Dyskinesia	1 (1.6)	0 (0)	1 (11)	0 (0)	0 (0)	0 (0)
		11. Myoclonias	0 (0)	0 (0)	0 (0)	0 (0)	0 (0)	0 (0)
		12. Speech alterations	13 (20.9)	5 (71.4)	4 (44.4)	3 (17.6)	1 (4)	0 (0)
		13. Memory deficit	2 (3.2)	1 (14.2)	0 (0)	0 (0)	1 (4)	0 (0)
		14. Visual/auditory hallucinations	2 (3.2)	1 (14.2)	0 (0)	1 (5.8)	0 (0)	0 (0)
		15. Seizures	1 (1.6)	0 (0)	1 (11.1)	0 (0)	0 (0)	0 (0)
		16. Neck stiffness	3 (4.8)	1 (14.2)	1 (11.1)	0 (0)	1 (4)	0 (0)
		17. Photophobia	8 (12.9)	2 (28.5)	2 (22.2)	3 (17.6)	1 (4)	0 (0)
		18. Altered muscle tone	2 (3.2)	1 (14.2)	0 (0)	1 (5.8)	0 (0)	0 (0)
		19. Abnormal reflexes	2 (3.2)	1 (14.2)	0 (0)	0 (0)	1 (4)	0 (0)
	Non-specific symptoms	20. Apathy	11 (17.7)	5 (71.4)	2 (22.2)	1 (5.8)	3 (12)	0 (0)
		21. Lack of appetite	12 (19.3)	2 (28.5)	1 (11.1)	5 (29.4)	4 (16)	0 (0)
		22 Asthenia	26 (41.9)	4 (57.1)	2 (22.2)	6 (35.2)	14 (56)	0 (0)
		23. Changes in sleep/wake rhythm	3 (4.8)	1 (14.2)	0 (0)	1 (5.8)	1 (4)	0 (0)
		24. Headache	34 (54.8)	3 (42.8)	4 (44.4)	10 (58.8)	17 (68)	0 (0)
		25. Limb or trunk pain	11 (17.7)	3 (42.8)	2 (22.2)	3 (17.6)	3 (12)	0 (0)
		26. Paresthesias/anesthesia	1 (1.6)	0 (0)	0 (0)	1 (5.8)	0 (0)	0 (0)
EEG		Focal delta slow activity	13 (20.9)	4 (57.1)	5 (55.5)	4 (23.5)	0 (0)	0 (0)
		Diffuse delta slow activity	7 (11.3)	3 (42.8)	3 (33.4)	1 (5.8)	0 (0)	0 (0)
		Focal epileptic abnormalities in sleep	15 (24.1)	7 (100)	6 (66.6)	2 (11.7)	0 (0)	0 (0)
		Normal	6 (9.7)	0 (0)	0 (0)	2 (11.7)	4 (12)	0 (0)
		Not performed	36 (58)	0 (0)	1 (11.1)	10 (58.8)	21 (84)	4 (100)
MRI		Altered	3 (4.8)	3 (42.8)	0 (0)	0 (0)	0 (0)	0 (0)
		Normal	7 (11.3)	2 (28.5)	4 (44.4)	1 (5.8)	0 (0)	0 (0)
		Not performed	52 (83.8)	2 (28.5)	5 (55.5)	16 (94.1)	25 (100)	4 (100)
CSF		Altered	0 (0)	0 (0)	0 (0)	0 (0)	0 (0)	0 (0)
		Normal	6 (9.5)	6 (85.7)	0 (0)	0 (0)	0 (0)	0 (0)
		Not performed	56 (90.5)	5 (71.4)	6 (66.6)	17 (100)	24 (96)	4 (100)
Steroid treatment		No	4 (6.5)	0 (0)	0 (0)	1 (5.8)	1 (4)	2 (50)
		2 mg/kg	36 (58)	1 (14.2)	5 (55.5)	13 (76.4)	18 (72)	1 (25)
		10 mg/kg	13 (21)	0 (0)	2 (22.2)	5 (29.4)	5 (20)	1 (25)
		30 mg/kg	9 (14.5)	6 (85.7)	2 (22.2)	0 (0)	1 (4)	0 (0)

*EEG, electroencephalography; MRI, magnetic resonance imaging; CSF, cerebrospinal fluid.*

*Signs of altered mental status* were observed in 29 patients (46.7%), of variable severity and in different combinations. Irritability or/agitation was observed in 18 patients (29%), and altered state of consciousness in 16 (25.8%). With regard to behavioral changes, emotional lability/impulsivity was seen in 10 patients (16.1%) and mood deflection/anxiety (e.g., inconsolable crying) in nine (14.5%).

*Focal neurological signs* were observed in 22 patients (35.4%), in different combinations. Changes in speech were reported in 13 children (20.9%), gait disturbances in eight (12.9%), photophobia in eight (12.9%), abnormal eye movements in three (4.8%), neck stiffness in three (4.8%), muscle tone alterations in two (3.2%), memory impairment in two (3.2%), visual/auditory hallucinations in two (3.2%), and abnormal reflexes in two (3.2%); facial asymmetries, dyskinesia, and seizures were each observed in one patient (1.6%). Hemiparesis and flaccid paralysis were not observed.

*Non-specific symptoms* were reported in 54/62 patients (87%), in different combinations. headache in 34 (54.8%), asthenia in 26 (41.9%), lack of appetite in 12 (19.3%), apathy in 11 (17.7%), limb or trunk pain in 11 (17.7%), sleep/wake rhythm changes in three (4.8%), and anesthesia or paresthesia in one (1.6%).

#### Electroencephalographic

Electroencephalographic (EEG) was performed in 26/62 children (42%): 20/62 (32.2%) showed abnormalities, particularly high-amplitude delta slow activity, which was diffuse in seven patients (11.3%) and observed predominantly over the posterior regions in the other 13 (20.9%). During sleep, posterior delta biphasic complexes and focal bilateral anterior theta rhythmic discharges were detected in 15 patients (24.1%).

#### Magnetic Resonance Imaging

Ten patients underwent brain Magnetic Resonance Imaging (MRI). In three (4.8%), a median, oval-shaped cytotoxic lesion was detected in the splenium of corpus callosum. In all cases, the cytotoxic lesion of the corpus callosum (CLOCC) was characterized by T2-weighted hyperintensity and restricted diffusion on diffusion weighted imaging (DWI) and apparent diffusion coefficient (ADC) maps. Follow-up MRI examination (after 1 week in one patient and 2 weeks in the other two) showed complete resolution of the signal abnormality. No other alteration was detected.

#### Cerebro Spinal Fluid

Cerebro Spinal Fluid (CSF) analysis was performed in six patients (9.5%). All the examinations were normal. SARS-CoV-2 was not detected.

#### Neurological Profiles

**FIGURE 2:**
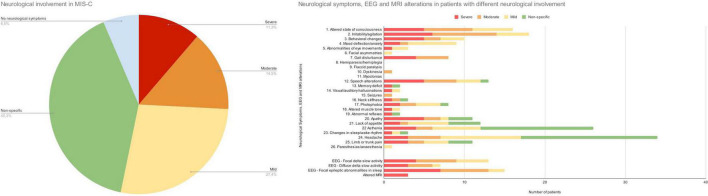
Distribution of neurological involvement in MIS-C. Neurological symptoms and EEG and MRI alterations in patients with different profiles of neurological involvement.

The distribution of neurological signs and symptoms according to the severity of neurological involvement is described in Figure 2.

#### Encephalitis

This group comprised 16 children: seven with severe and nine with moderate encephalopathy.

**FIGURE 3:**
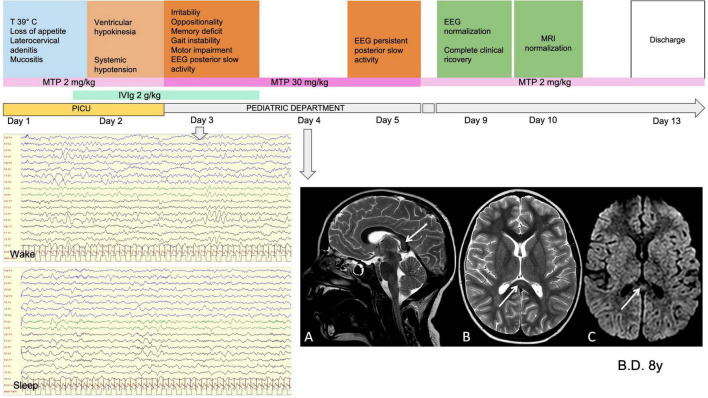
Clinical, MRI and EEG findings in representative patients with focal EEG alterations. Male, 8 years old. MISC presentation characterized by fever (T 39^°^C), systemic symptoms, ventricular hypokinesia and neurological symptoms (irritability, oppositionality, memory deficit gait instability, motor impairment). WBC 16460 (10^A^3/mmc) (N 14579, L 1030); PLTs 152 (10^A^3/mmc); Hb 10.1 (g/dL); CRP 189.6 (mg/l); Ferritin B29 (mcg/l); D-dimer 1114 (mcg/l); NT-ProBNP 15979 (ng/l); TnT 28 (ng/l). On day 1 he was given Mg (2 g/kg for 2 days) and on day 2 was started on high-dose MTP (30 mg/kg for 3 days). MRI performed on day 4 **(A)**. Sagittal, **(B)** Axial T2-weighted images show slight focal median hyperintense lesion in the splenium of corpus callosum (arrows). **(C)** Axial diffusion-weighted image shows reduction of proton diffusivity at the site of the lesion (arrow). EEG performed on day 3 **(D)**. High-amplitude delta slow activity prevalent in the posterior regions **(E)**. Slow activity during sleep with preserved spindles. Clinical and EEG normalization was observed on day 7 and normalization of MRI on day 9. CK, creatine kinase; WBC, white blood cells; N, neutrophils; L, lLymphocytes; PLTs, platelets; Hb, hemoglobin; CRP, C-reactive protein; NT-ProBNP, amino-terminal fragment of the brain natriuretic peptide; TnT, troponin T; IVIg, intravenous immunoglobulin; MTP, methylprednisolone; MRI, magnetic resonance imaging; CC, corpus callosum; EEG, electroencephalography; PICU, pediatric intensive care unit admission.

**FIGURE 4:**
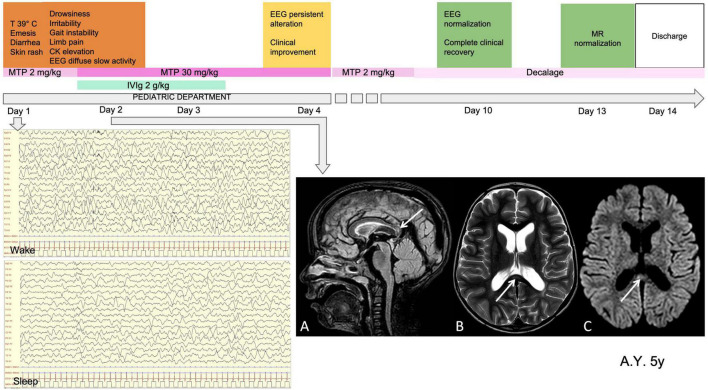
Clinical, MRI and EEG findings in a representative patient with diffuse EEG alterations. Male, 5 years old. MISC presentation characterized by fever (T 39^°^C), gastrointestinal involvement, skin rash, neurological symptoms (drowsiness, irritability, gait instability, limb pain) and CKelevation. Laboratory data at onset WBC 3420 (10^A^3/mmc) (N 1810, L 1470); PLTs 224 (10^A^3/mmc); Hb 11.9 (g/dL); CRP 33 (mg/l); ferritin 829 (mcg/l); D-dimer 12077 (mcg/l); NT-ProBNP 1982 (ng/l); TnT 820 (ng/l). On day 1 he was given IVIg (2g/kg for 2 days) and on day high-dose steroid treatment was started (MTP 30 mg/kg for 3 days). MRI performed on day 2 **(A)**. Sagittal FLAIR image, **(B)** Axial T2-weighted images show slight focal median hyperintense lesion in the splenium of corpus callosum (arrows). Parenchymal signal abnormality and thickness reduction can be observed in the peritrigonal white matter on the left side in **(B)**. **(C)** Axial diffusion- weighted image shows reduction of proton diffusivity at the site of the CC lesion (arrow). EEG performed on day 1 **(D)**. Diffuse high-amplitude delta slow activity **(E)**. Loss of organization of sleep activity, with high-amplitude delta slow waves. Clinical and EEG normalization was observed on day 10 and normalization of MRI on day 13. CK, creatine kinase; WBC, white blood cells; N, neutrophils; L, lymphocytes; PLTs, platelets; Hb, hemoglobin; CRP, C-reactive protein; NT-ProBNP, amino-terminal fragment of the brain natriuretic peptide; TnT, troponin T; IVIg, intravenous immunoglobulin; MTP, methylprednisolone; MRI, magnetic resonance imaging; CC, corpus callosum; EEG, electroencephalography; PICU, pediatric intensive care unit admission.

The children with severe encephalopathy (7/62, 11.3%) displayed both focal and diffuse neurological signs. Within this subgroup, we observed severe irritability (85.7%), mood deflection (28.5%), and drowsiness (71.4%), variably associated with apathy (71.4%), headache (42.8%), meningism (14.2%), photophobia (28.5%), oculomotor apraxia (14.2%), gait disorder (57.1%), pain (42.8%), and slow, “whiny” and repetitive speech with reduced verbal output and preserved comprehension (71.4%). One patient presented a generalized tonic-clonic seizure during fever. Wake EEG at the onset of the neurological symptoms was dominated by high-amplitude delta slow activity, diffuse (42.8%) or recorded predominantly over the posterior regions (57.1%). During sleep, posterior delta biphasic complexes and focal bilateral anterior theta rhythmic discharges were detected. When encephalitis was confirmed, these children received treatment with high-dose MTP. The symptoms peaked in 2–3 days and thereafter showed a dramatic improvement. EEG usually improved markedly by 10 days, with progressive reorganization of background activity. A normal EEG was usually recorded between 15 and 24 days from the start of steroid therapy. In three of the seven patients in this subgroup, brain MRI documented CLOCCs; in the others, it was normal. CSF analysis was carried out in 6/7 children, giving normal results. Figures 3, 4 show the clinical and EEG changes recorded over time in two representative patients with focal (Figure 3) and diffuse EEG alterations (Figure 4).

The children with moderate encephalopathy (9/62, 14.5%) showed mild symptoms of diffuse encephalopathy, namely mild irritability (88.8%), drowsiness (66.6%), mood deflection (11.1%), and headache (44.4%). Within this subgroup, EEG documented focal (55.5%) or diffuse delta waves (33.3%), high-amplitude biphasic delta waves in sleep, and/or focal spikes. All these patients recovered within 5–8 days from the start of steroid therapy, and the EEG performed between days 15 and 21 was normal. Brain MRI was performed in three of the children with moderate encephalopathy and was normal in all of them. None underwent CSF analysis.

#### Mild Neurological Involvement

This group comprised 42 children: 17 with mild clinical involvement and 25 with non-specific neurological signs.

In the subgroup of children with mild clinical involvement (17/62, 27.4%), we observed mild mood deflection and anxiety (35.2%), mild irritability (23.5%), drowsiness (29.4%), headache (58.8%), and asthenia (35.2%).

Among the children with non-specific neurological signs (25/62, 40.3%), the main complaints were asthenia (56%), limb or trunk pain (12%), headache (68%), usually worse at the peak of fever, apathy (12%), lack of appetite (16%), sleep/wake rhythm changes (4%), and mild photophobia (4%).

#### Systemic Dysfunction and Its Relationship With Neurological Profiles

Table 2 sets out the data on systemic dysfunction in these patients, namely the number of systems involved, the rates of cardiac involvement and of PICU admission, laboratory data and steroid therapy doses. The data are presented both for the entire sample and by severity of neurological involvement.

In 62.5% (10/16) of the patients with encephalitis, and in 50% (23/46) of those with either mild or no neurological involvement, more than two organs or systems were affected, excluding the neurological system.

Cardiac involvement (LVFE < 45%) was documented in 31.25% (5/16) of the children with encephalitis and in 30.4% (14/46 patients) of those with either mild or no neurological involvement. Respiratory involvement was observed in 66.1% (44/62) and abdominal involvement in 83.9% (52/62), with no statistically significant difference between the two severity groups. No patient showed acute kidney injury (AKI).

Seven of the 16 patients (43.75%) with encephalitis and 29/46 (63%) of those with either mild or no neurological involvement were admitted to the PICU. Shock was reported in 16.1% (10/62); again, no statistical difference was found between the two groups.

Laboratory data (Table 1) showed elevated inflammatory markers (C-reactive protein, fibrinogen, ferritin, D-dimer), neutrophilia with lymphopenia, normal platelet counts and hemoglobin levels, and increased cardiac biomarkers in both groups.

No statistically significant differences in demographic data, comorbidities, organ or system involvement, and hospitalization or laboratory data were found between the two comparison groups. Only patient age differed. In fact, patients with encephalitis reported a lower median age compared with the other group (5 years vs. 10 years, *p* = 0.00048).

## Discussion

This study describes the spectrum of neurological involvement in a sample of 62 children with MIS-C admitted to a single tertiary pediatric department between October 1, 2020 and March 31, 2022.

Neurological signs and symptoms were present in 93.5% of the patients. Headache, of varying duration and severity, was the most commonly reported symptom, occurring in over half of the subjects, followed by asthenia. Although in themselves and if present in isolation these symptoms are poorly specific and not indicative of neurological involvement, we found that they were rarely isolated; for this reason, we believe they should be considered red flags of possible neurological involvement, and therefore that their presence should prompt a more detailed evaluation of the clinical picture. Approximately half of the sample showed clear signs of altered mental status, mainly drowsiness and irritability, associated with apathy and mood and behavioral changes. Overall, these disturbances, severe and pervasive in most cases, made the children “unrecognizable” to their parents. Parents reported that their children would swing between states of apathy-drowsiness and fits of anger and irritability, for no apparent reason. With the exception of speech and gait abnormalities, observed in around a fifth and a sixth of the children, respectively, focal neurological signs were observed in very few children. No child showed acute motor deficits or signs of peripheral nervous system involvement. Just one had a generalized seizure during fever.

The incidence of neurological involvement in our sample was found to be much higher than the rates of up to around 50% reported in the literature (3, 4, 15, 16). This difference is very likely due to the fact that the patients in the present study were all assessed, at admission or shortly afterward, by a child neurologist who was able to detect and evaluate even mild neurological signs and symptoms, and it suggests that specialist assessment of neurological aspects should be routinely included in the workup of patients with MIS-C.

Although very frequent, the neurological involvement observed in our sample was not serious in most cases: around two thirds of the affected children had mild and short-lasting symptoms. This is in line with the findings of Larovere et al., who reported transient symptoms in 88% of their subjects with neurological involvement (17).

In children with encephalitis, the clinical picture was dominated by encephalopathy and EEG abnormalities very similar to those previously reported in the first cases we observed (4). Indeed, in the present study, too, the EEG recordings showed well-defined characteristics, confirming that EEG plays a valuable role in the diagnosis of encephalitis in children with MIS-C and altered mental status. At the onset of the neurological symptoms, EEG typically showed absence of physiological organization, particularly on awake recordings, which were dominated by high-amplitude delta slow activity, diffuse or predominant over the posterior regions. During sleep, posterior delta biphasic complexes and focal bilateral anterior theta rhythmic discharges were detected. The symptoms peaked in 2 or 3 days and showed a dramatic improvement after high-dose MTP treatment. Neurological assessment at discharge was usually normal. EEG background activity improved from the first week, and normalized within 2 or 3 weeks.

Among the patients with encephalitis, three showed diffuse abnormal T2-weighted hyperintensity and restricted diffusion involving the white matter and genu or splenium of corpus callosum on MRI. CLOCCs have already been described as a potential neuroradiological presentation of SARS-CoV-2 infection in adults (18, 19) and, in the context of inflammatory multisystem syndrome, temporally associated with SARS-CoV-2 exposure in children (20). Our cases with callosal lesions expand the series already described and confirm that CLOCCs could be a neurological complication of SARS-CoV-2 exposure in children. In the literature, CLOCCs are reported to be secondary lesions associated with different entities, including infections, all sharing specific features: high levels of cytokines and extracellular glutamate (21). It has been hypothesized that the corpus callosum shows selective vulnerability to cytokine storms due to its high density of cytokine and glutamate receptors (21); a cytokine storm seems to be the cause of the callosal neuron and microglia dysfunction that allows T cells to breach the BBB, causing inflammation and intramyelinic edema (22). The rapid resolution of the neuroradiological picture observed in our cases supports this hypothesis and suggests that immunotherapy can play a key role in the treatment of this condition.

The children with encephalitis (median age 5 years) were younger than those with mild or no neurological impairment (median age 10 years), an aspect that points to a possible, and as yet undescribed, age dependence of the neurological clinical pictures associated with MIS-C. Although neurological involvement (sterile meningoencephalitis) has been reported in in Kawasaki disease, which mainly affects preschool-age children and shows a known clinical overlap with MISC in very young children, we found the neurological phenotype in this latter group to be more severe and more complex than what has been described in patients with Kawasaki disease (23). Indeed, while greater severity of MIS-C has been described in children > 5 years of age, to date no age differences have been reported in the literature with regard to neurological involvement (24).

The other demographic data analyzed — sex and ethnicity — showed no significant differences. In comparison with literature data (25), our sample showed significantly increased frequency of comorbid neurological or immune system disorders. This might be due to the fact that our diagnostic protocol, being designed with a view to multidisciplinary management of these patients, allowed us to collect a larger and broader body of data than are provided by the retrospective studies present in the literature (25). In our study, the presence of these comorbidities was not associated with a significantly increased risk of developing neurological complications, but this observation needs to be verified over time in larger samples.

The severity of systemic involvement and the number of organs and systems (including cardiac and gastrointestinal) affected, as well as the hospitalization rates and patterns of laboratory data, were found to be similar in children with severe neurological involvement compared with what was observed in children with mild or no neurological involvement. In other words, encephalitis can be documented even in children with moderate changes in inflammatory indices and without severe systemic involvement, including cardiac involvement. This observation is in contrast to what has thus far been reported in the literature, in which more severe neurological involvement was associated with more extreme inflammation. Overall, our data support the need for neurological assessment of children with MIS-C (especially those younger than 10 years), regardless of the degree of cardiac involvement and of alteration of the inflammatory indices. It seems, in fact, that neurological involvement can be independent of cardiac and systemic involvement. Clarification, over time, of the reasons for this dissociation will allow the development of better-targeted diagnostic and therapeutic protocols.

In this regard, it should also be emphasized, in line with what has already been reported in the literature (3), that the most frequent symptoms — headache, asthenia, signs of altered mental status — are not particularly specific, and can certainly also have non-neurological causes. However, our experience and careful clinical evaluations have shown us that they rarely occur in isolation and seem to be a very sensitive indicator of more serious neurological involvement.

The findings here reported confirm, in a larger sample, what we have already observed in a previous, smaller case series, namely that children with MIS-C may present postinfectious autoimmune-mediated encephalitis that, probably the expression of immune system overactivation and of a cytokine storm, disappears in response to immunomodulatory therapy (5). They also confirm that these clinical pictures, if diagnosed and treated promptly, regress without leaving neurological sequelae (5). Clinical pictures similar to these have been described in the literature in children with SARS-CoV-2 infection, both with (MIS-C) and without multiple organ involvement: in these cases, too, the effectiveness of high-dose steroid therapy was emphasized (4, 12). However, this observation does not exclude the possibility of future sequelae. Long-term follow-up is therefore needed to evaluate effects of the infection on cognition and development.

To conclude, it is worth reflecting on the therapeutic implications of our observations in these patients.

Generally speaking, while the onset of MISC observed in our study resembled the onset symptoms described in cases reported in the literature, the clinical evolution of our patients was found to be relatively favorable (17). Early multidisciplinary diagnosis and the immediate use of immunomodulatory and anticoagulant therapies, as well as the inotropic support given to patients with severe cardiac dysfunction or with capillary leaks, certainly played a role in preventing other organ damage, such as AKI (26).

Even though our patients were hospitalized, on average, for longer than cases reported in the literature—12 days vs. 5 days (25)—the duration of their PICU stays was in line with what has been reported in other series. Our patients’ longer overall stays in the pediatric department were not linked to slower resolution of the symptoms, but rather to a deliberate policy choice on the part of our institution, which, in view of possible pandemic-related difficulties in ensuring adequate home observation, preferred to keep post-acute patients monitored in a protected environment for longer. We cannot exclude the possibility that this prolonged observation reduced the risk of some complications in the post-acute phase.

Systemic aspects apart, this approach probably also affected the overall neurological outcome. In fact, none of the children in our study displayed any of the other clinical pictures falling within the broad spectrum of severe neurological manifestations related to SARS-CoV-2 infection and MIS-C reported in the literature, and associated with worse long-term outcomes, namely ischemic or hemorrhagic stroke, demyelination, acute fulminant cerebral edema, and Guillain-Barré syndrome (3, 10). Indeed, it seems likely that the use of IVIG and steroids (administered at variable doses, according to severity) led to rapid resolution of the encephalitic symptoms, and that low-molecular-weight heparin and therapeutic-dose anticoagulant treatment reduced the risk of thromboembolic complications. This hypothesis, if confirmed in larger populations of patients, could strengthen the argument for the implementation of a more aggressive and broad-spectrum therapeutic approach in children with MIS-C.

## Conclusion

Neurological involvement in children with MIS-C is frequent but not serious in most cases: the majority of affected children had mild and short-lasting symptoms. A quarter of the children showed acute immune-mediated encephalitis with rapid-onset encephalopathy, focal neurological signs, and EEG slowing. This clinical picture disappeared with immunomodulatory therapy. The severity of the neurological picture seemed to be related to age, but not to the degree of multiorgan involvement and inflammation. Neurological assessment allowed timely diagnosis and treatment.

This study demonstrates the importance of including neurological evaluation in the diagnostic workup of MIS-C, regardless of clinical presentation, organ involvement, comorbidities, inflammatory indices, and laboratory data; this applies particularly in children younger than 10 years. In our sample we observed a favorable outcome in all cases, which can probably be linked to the prompt steroid and IVIG treatment administered in all patients.

## Milan MIS-C Study Group

Sara Olivotto^1^, Eleonora Basso^2^, Rossella Lavatelli^2^, Roberto Previtali^2^, Laura Parenti^1^, Gianvincenzo Zuccotti^2,3^, Laura Fiori^3^, Elena Zoia^4^, Anna Camporesi^4^, Veronica Diotto^4^ and Mirko Gambino^3^

^1^Pediatric Neurology Unit, V. Buzzi Children’s Hospital, ASST Fatebenefratelli Sacco, Milan, Italy.

^2^Department of Biomedical and Clinical Science, University of Milan, Milan, Italy.

^3^Department of Pediatrics, V. Buzzi Children’s Hospital, ASST Fatebenefratelli Sacco, Milan, Italy.

^4^Pediatric Intensive Care Unit, V. Buzzi Children’s Hospital, ASST Fatebenefratelli Sacco, Milan, Italy.

## Data Availability Statement

The raw data supporting the conclusions of this article will be made available by the authors, without undue reservation.

## Ethics Statement

The studies involving human participants were reviewed and approved by the Comitato etico Milano Area 1. Written informed consent to participate in this study was provided by the participants’ legal guardian/next of kin.

## Author Contributions

SMB had full access to all the data in the study and takes responsibility for the integrity of the data, the accuracy of the data analysis, and acquisition of clinical data. SMB and SM contributed to the conception, design, and acquisition of clinical data. LS, PC, ARD, and AG contributed to acquisition, analysis, interpretation of neurological data, and acquisition of clinical data. LL, AV, SM contributed to acquisition, analysis, interpretation of cardiological data, and acquisition of clinical data. CD contributed to acquisition, analysis, interpretation of neuroradiological data, and acquisition of clinical data. SMB, LS, PC, ARD, CD, and SM contributed to drafting of the manuscript and acquisition of clinical data. SMB, SM, and PV contributed to critical revision of the manuscript for important intellectual content and acquisition of clinical data. PC and LL contributed to statistical analysis and acquisition of clinical data. Milan MIS-C Study Group contributed to acquisition of clinical data. All authors contributed to the article and approved the submitted version.

## Conflict of Interest

The authors declare that the research was conducted in the absence of any commercial or financial relationships that could be construed as a potential conflict of interest.

## Publisher’s Note

All claims expressed in this article are solely those of the authors and do not necessarily represent those of their affiliated organizations, or those of the publisher, the editors and the reviewers. Any product that may be evaluated in this article, or claim that may be made by its manufacturer, is not guaranteed or endorsed by the publisher.
